# Excitatory Impact of Dental Occlusion on Dorsal Motor Nucleus of Vagus

**DOI:** 10.3389/fncir.2021.638000

**Published:** 2021-03-12

**Authors:** Xin Liu, Minghong Shi, Haotian Ren, Mianjiao Xie, Chunkui Zhang, Dongmei Wang, Xiaodong Liu, Jinlian Li, Meiqing Wang

**Affiliations:** ^1^State Key Laboratory of Military Stomatology & National Clinical Research Center for Oral Diseases & Shaanxi International Joint Research Center for Oral Diseases, Department of Oral Anatomy and Physiology, School of Stomatology, The Fourth Military Medical University, Xi'an, China; ^2^School of Stomatology, The Third Affiliated Hospital of Xinxiang Medical University, Xinxiang, China; ^3^Department of Stomatology, Changhai Hospital, The Second Military Medical University, Shanghai, China; ^4^Department of Anatomy, Histology and Embryology and K.K. Leung Brain Research Centre, The Fourth Military Medical University, Xi'an, China

**Keywords:** trigeminal mesencephalic nucleus, dorsal motor nucleus of vagus nerve, unilateral anterior crossbite, occlusion, vesicular glutamate transporter 1, glucagon, insulin, blood glucose

## Abstract

Neurons in the trigeminal mesencephalic nucleus (Vme) have axons that branch peripherally to innervate the orofacial region and project centrally to several motor nuclei in brainstem. The dorsal motor nucleus of vagus nerve (DMV) resides in the brainstem and takes a role in visceral motor function such as pancreatic exocrine secretion. The present study aimed to demonstrate the presence of Vme–DMV circuit, activation of which would elicit a trigeminal neuroendocrine response. A masticatory dysfunctional animal model termed unilateral anterior crossbite (UAC) model created by disturbing the dental occlusion was used. Cholera toxin B subunit (CTb) was injected into the inferior alveolar nerve of rats to help identify the central axon terminals of Vme neurons around the choline acetyltransferase (ChAT) positive motor neurons in the DMV. The level of vesicular glutamate transporter 1 (VGLUT1) expressed in DMV, the level of acetylcholinesterase (AChE) expressed in pancreas, the level of glucagon and insulin expression in islets and serum, and the blood glucose level were detected and compared between UAC and the age matched sham-operation control mice. Data indicated that compared with the controls, there were more CTb/VGLUT1 double labeled axon endings around the ChAT positive neurons in the DMV of UAC groups. Mice in UAC group expressed a higher VGLUT1 protein level in DMV, AChE protein level in pancreas, glucagon and insulin level in islet and serum, and higher postprandial blood glucose level, but lower fasting blood glucose level. All these were reversed at 15-weeks when UAC cessation was performed from 11-weeks (all, *P* < 0.05). Our findings demonstrated Vme–DMV circuit *via* which the aberrant occlusion elicited a trigeminal neuroendocrine response such as alteration in the postprandial blood glucose level. Dental occlusion is proposed as a potential therapeutic target for reversing the increased postprandial glucose level.

## Introduction

The periodontal biomechanical message is mediated by trigeminal mesencephalic nucleus (Vme) which resides in the brainstem. The cell bodies of the neurons in Vme form a mediolaterally narrow band of neurons located across the entire rostro-caudal axis of the midbrain (Lazarov, 1996). This anatomic feature makes it possible for the projections of neurons in the Vme to target many other neurons in the brainstem. The neurons of Vme are pseudo-monopolar neurons, the peripheral processes of which extend to the periodontal ligament through the inferior alveolar nerve, and the central processes extend to many motor neurons such as motor neurons of trigeminal motor nucleus, facial nucleus, hypoglossal nucleus, and spinal nucleus of the accessory nerve. Hence, by injecting CTb into alveolar nerve, the CTb positive terminals could be observed at the sites where the central processes of neurons in Vme extend to (Zhang F. X. et al., 2018). In recent, we developed an experimental unilateral anterior crossbite (UAC) rodent model which induced osteoarthritic lesions in the temporomandibular joints (Zhang et al., 2015, 2016, 2019; Zhang J. et al., 2018; Yang et al., 2020). By using the reported CTb tract tracing methods (Zhang F. X. et al., 2018) combined with the detection of the protein expression level of acetylcholinesterase (AChE) in masseters, stapedius, lingualis, and sternocleidomastoid muscles, the excitatory impact of UAC on trigeminal motor nucleus, facial nucleus, hypoglossal nucleus, and spinal nucleus of the accessory nerve were demonstrated (Liu et al., 2017, 2018).

The dorsal motor nucleus of vagus nerve (DMV), an important visceral motor nucleus located in brain stem, has been acknowledged as the largest source of parasympathetic preganglionic neurons within the lower brainstem (Huang et al., 1993). It is evident that the DMV is the site of origin of vagal efferent neurons that innervate both the endocrine and the exocrine pancreas (Berthoud and Powley, 1991; Jansen et al., 1997). Several neurophysiological studies have shown that stimulation of the DMV activates both endocrine and exocrine secretion *via* a cholinergic pathway, which elicits significant increases in insulin and glucagon secretion (Mussa et al., 2011; Mussa and Verberne, 2013). Both insulin and glucagon take a role in control of blood glucose level. There is negative report which indicates that the thoroughly mastication increased the endogenous glucagon-like peptide 1, compared with usual mastication, without affecting the concentrations of blood glucose or serum insulin (Sonoki et al., 2013). However, mastication frequency, eating speed, and eating duration have been reported to impact on incretin secretion (Fujiwara et al., 2019). Stimulation by mastication alone triggers insulin secretion and patients with malocclusion tend to show increased insulin resistance (Hashimoto et al., 2011). It is then attractive to detect whether there is Vme–DMV circuit *via* which UAC has an impact on blood glucose levels.

Within the central nervous system, vesicular glutamate transporters (VGLUTs) are essential for signal output through the condensation of glutamate into vesicular constituents for subsequent exocytotic release upon stimulation (Bai et al., 2001). Vme neurons have been indicated to be glutaminergic (Lazarov, 2000). In Vme, the vesicular glutamate transporter 1 (VGLUT1) takes the major role of mediating the vesicular secretion of glutamate (Lazarov, 2000). Thus, increase of VGLUT1 mRNA in Vme which implies an active local production of VGLUT1 is generally used as a sign of excitation of Vme neurons (Fremeau et al., 2004). The synthetic VGLUT1 protein is transported from the cell bodies to the peripheral endings and central axons. VGLUT1 protein is then located at the sites where the peripheral endings and central axons of the Vme neurons extend. By detecting the VGLUT1 protein expression at the targeted sites, the activation of Vme can be confirmed (Pang et al., 2006). Acetylcholine is the main neurotransmitter of motor neurons. Choline acetyltransferase (ChAT) is the key enzyme in acetylcholine synthesis (Armstrong et al., 1983) and is therefore usually taken as a marker of motor neurons. Acetylcholinesterase (AChE) is a secretive carboxylesterase that can rapidly hydrolyze the neurotransmitter acetylcholine at cholinergic synapses, which is important for the correct function of the nervous system (Dvir et al., 2010). The upregulated expression level of AChE at the target sites could be taken as an excitatory sign of acetylcholinergic neurons innervate that sites. By testing the expression of VGLUT1 mRNA of Vme neurons in UAC treated rats *via in situ* hybridization histochemistry and real-time PCR (Liu et al., 2017), and by detecting VGLUT1 protein expression in motor neurons of trigeminal motor nucleus, facial nucleus, hypoglossal nucleus, and spinal nucleus of the accessory nerve, the impact of UAC on functions of masseter, stapedius, lingualis, and sternocleidomastoid muscles, respectively, were verified (Liu et al., 2017, 2018). Using the same method, the impact of UAC on DMV could be tested.

In this study, to demonstrate the projection of Vme to DMV, we injected cholera toxin B subunit (CTb) into the inferior alveolar nerve of rats as we previously reported (Liu et al., 2017) and tested its expression around the neurons in DMV. To detect whether dental occlusion has an excitatory impact on Vme–DMV pathway, we adopted UAC model. Co-expression of CTb and VGLUT1 protein in DMV was detected to illustrate the activation of the neurons in DMV by the UAC. The VGLUT1 mRNA level in Vme, the protein level of VGLUT1 and ChAT in DMV, AChE in pancreas, and insulin and glucagon in both pancreas and serum were tested, and the level of blood glucose in serum was measured to indicate the activation of DMV by the UAC. The purpose was to demonstrate the presence of Vme–DMV circuit, activation of which would elicit trigeminal neuroendocrine response of blood glucose level.

## Materials and Methods

### Animals and Grouping

**Figure 1:**
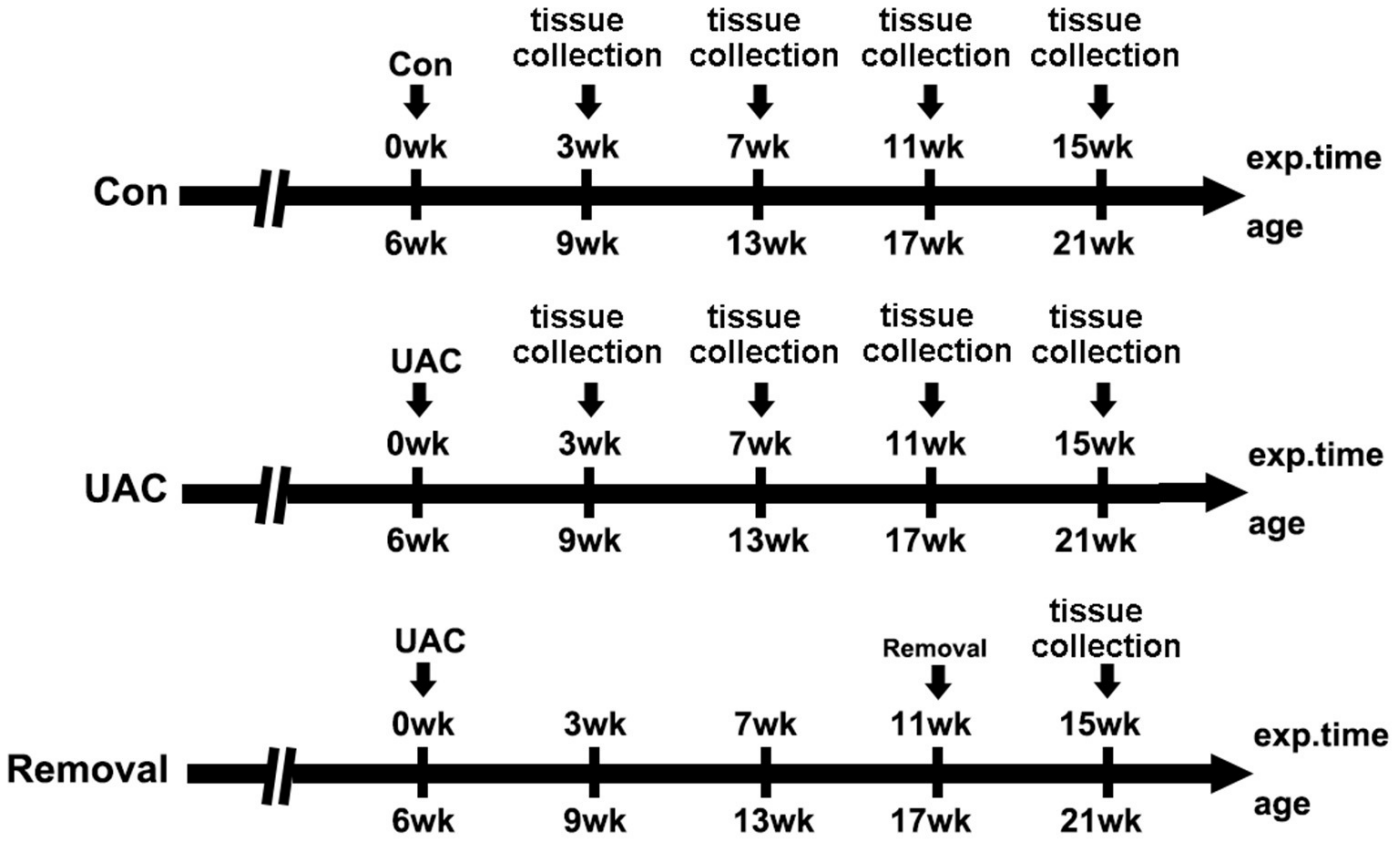
The timeline of the entire experiment. exp. time, experimental time point; age, the natural age of the mice; Con, control group; UAC, unilateral anterior crossbite prosthesis; and Removal, removal of the unilateral anterior crossbite prosthesis; exp., experimental.

Twelve female 6-weeks old Sprague-Dawley rats (130–160 g) and fifty-four 6-weeks old C57 mice (15–20 g) were provided by the Animal Center of Fourth Military Medical University. All procedures and animal care were approved by the University Ethics Committee and were performed according to institutional guidelines. The 12 rats were divided into UAC and control groups equally (*n* = 6) and used for transganglionic tract tracing. The 54 mice were randomly divided into the UAC group and sham control group (*n* = 6) at four time points, that were 3-, 7-, 11-, and 15-weeks, and removal group (*n* = 6) in which the UAC was removed after 11-weeks onset and the animals were sampled at 15-weeks as we previously reported (Zhou et al., 2020). The timeline of the entire experiment were presented in Figure 1.

Details for the animal distribution in the experimental groups and subgroups were presented in Table 1.

**Table 1:** Distribution of the experimental animals in the subgroups.

**Group**	**Number of animals in each group (*N*)**	**Procedure**
Nerve tract tracing-UAC	6	Injection of CTb into the inferior alveolar nerve of the rat
Nerve tract tracing-Con	6	Injection of CTb into the inferior alveolar nerve of the rat
3-week UAC	6	1) Immunohistochemical staining and analysis of pancreas of mice 2) Brain tissue of mice for real-time quantitative PCR analysis 3) Brain and pancreas tissues of mice for WB analysis 4) Blood samples from orbital venous plexus for ELISA analysis in mice
3-week Con	6	
7-week UAC	6	
7-week Con	6	
11-week UAC	6	
11-week Con	6		
15 week UAC 15-week Con	6 6	Outside of the experiments in (1)–(4) above, Blood samples from caudal vein of mice for Oral glucose tolerance test (OGTT) on day 0, 3-week, 7-week, 11-week, and 15-week, respectively
15-week Removal	6	The UAC model was removed at the beginning of the 11th week after UAC application, similarly carry out experimental manipulations (1)–(4), and Oral glucose tolerance test (OGTT)

### UAC Application and Removal

Mice were anesthetized with sodium pentobarbital (40 mg/kg, intraperitoneal injection, i.p.) to reduce tracheal secretions. Metal tubes were affixed to the left-side incisors to establish UAC as we previously described (Liu et al., 2014; Lu et al., 2014). Briefly, a metal tube, 1.5 mm long, was made of a pinhead (inner diameter = 0.61 mm, thickness = 0.3 mm) to fit to the left-side maxillary incisor. The mandibular tube was curved to form a 135° labially inclined occlusal plate to create a crossbite relation with the maxillary-tubed incisor. The tubes were carefully bonded with zincphosphate cement under anesthesia using intraperitoneal injection of 1% pentobarbital and were checked every 2 days. No prosthesis fell off during the experimental period. Using the similar methods, tubes with larger diameters were used for rats to fit the large incisors (Zhang et al., 2016; Zhang J. et al., 2018). In the control group, the animals received the similar procedures, but no metal tubes were bonded. That meant the animals in the control group accepted a sham operation. In the Removal group, the UAC model was removed at the beginning of the 11th week after UAC application (Zhou et al., 2020). All the animals were fed with cylindrically shaped pressed food pellets. During the experiment, there was no significant difference in body weight and food intake.

### Lower Alveolar Nerve Injection of CTb Solution

Twelve adult female rats were anesthetized by intraperitoneal injection with 1% pentobarbital. As we previously reported (Liu et al., 2017), the left inferior alveolar nerve, which supplies the mandibular teeth, was carefully exposed at the level of the lower third molar *via* an extra-oral incision along the lower border of the mandible. A small bone window of about 3 × 3 mm was drilled in the lower jaw with a dental drill to expose the lower alveolar nerve. The water solution containing 1% CTb (Sigma, St Louis, MO, USA) of 6–8 μl was slowly injected into the lower alveolar nerve with a glass micropipette (internal tip diameter 15–20 μm) attached to a 10 μl Hamilton microsyringe (Hamilton Robotics, Reno, NV, USA). Cover the bone window and suture the masseter muscle and skin. The rats were allowed to survive for 5 days before sacrificial sampling.

### Oral Glucose Tolerance Test (OGTT)

For the measurement of glucose metabolism, blood glucose meter, and test strips (Yuehao720, Yuwell, China) were used for OGTT. All mice were deprived of food for 20 h before the testing. A glucose solution was given by weight, and blood samples were taken from the caudal vein before and at 10, 20, 30, 40, 60, 90, and 120 min after administration of the glucose solution. The read values of OGTT were used for comparison between groups.

### Glucagon and Insulin Test

Under anesthesia, blood samples from orbital venous plexus were kept at 4°C overnight. The supernatant was taken as serum for hormone test after centrifugation for 20 min at 1,000*g*. The operation was carried out in strict accordance with the instructions using the mouse glucagon and insulin ELISA kit (Elabscience, Wuhan, China). The glucagon and insulin kits were taken out of the refrigerator for 20 min before the experiment and balanced to room temperature. The working fluid was prepared according to the instructions and the serum samples were diluted five times.

Sample dilution of 50 μl were added to 96-well plate or standard and incubated at 37°C for 60 min. After five times of washing, the chromogenic solution was added evenly. At 37°C, the samples were incubated in the dark for 15 min. The absorbance (OD-value) of each well was measured at the wavelength of 450 nm after mixing with 50 μl termination solution. The test was performed within 15 min after the termination fluid was added. Standard curve equations and sample concentrations were calculated.

### Preparation of Brainstem Slice Samples

For histology, animals from the UAC and the control groups (*n* = 6) were euthanized with sodium pentobarbital (120 mg/kg, i.p.), and transcardially perfused with saline followed by 4% (w/v) paraformaldehyde in 0.1 M phosphate buffer (PB; pH 7.3) containing 4% (w/v) paraformaldehyde and 75% (vol/vol) saturated picric acid. The brain was carefully removed and post-fixed in the same fixative for 2 h and placed into 0.1 M PB containing 30% (w/v) sucrose overnight. The brain area containing the Vme, and DMV nuclei were extracted and cut into 25-μm-thick coronal sections by a Leica cryostat (Leica Biosystems, Heidelberger, Nussloch, Germany).

### Immunohistochemical Staining of Pancreas Tissues and Analysis

The mice were sacrificed after OGTT and hormone test. The pancreas was quickly removed and fixed in 4% paraformaldehyde for 24 h. After ethanol gradient dehydration and xylene immersion, the mice were embedded in paraffin, marked with clipping, and prepared for sections. Five-micron thick sagittal sections were used and randomly selected for Hematoxylin and Eosin (HE) staining. As previously described, a three-step avidin-biotin complex staining procedure was taken (Wang et al., 2014). The pancreas sample was performed using insulin polyclonal antibody (1:1,000, Abcam) and glucagon (1:1,000, Abcam) to detect the changes of insulin and glucagon expression in islets. The insulin and glucagon-positive cell area was stained orange and other negative cell area of islet was pale. HE-stained sections were analyzed under a light microscope (Leica DM2500, Wetzlar, Germany), and images were acquired using a Leica DFC 490 system (Leica Microsystems, Wetzlar, Germany). Using Photoshop software (Adobe Photoshop CS3, Adobe, CA, USA), the percentage of the insulin and glucagon-positive area relative to the whole area of the islet was calculated (*n* = 6), and the average value was used to represent the positive area percentage for each sample.

### Immunofluorescence Histochemical Staining of DMV and Analysis

Triple immunofluorescence staining of CTb, VGLUT1, and ChAT was conducted for CTb-injected rats. Sections containing DMV was processed for CTb, VGLUT1, and ChAT triple-immunofluorescence staining. Briefly, the sections were incubated at room temperature with a mixture of goat anti-CTb Ig (1:2,000; List Biological, Campbell, CA, USA), polyclonal rabbit anti-VGLUT1 Ig (1:500; Synaptic System, Gottingen, Germany), and mouse anti-ChAT Ig (1:500; Synaptic System) for 16–18 h. The sections were then incubated with a mixture of Alexa488-conjugated donkey anti-goat IgG (1:500; Millipore), Cy3-conjugated donkey anti-rabbit IgG (1:1,000; Millipore), and Alexa647-conjugated donkey anti-mouse IgG (1:500; Millipore) for 4 h at room temperature. Immunohistochemical controls were performed by omission of primary and secondary antibody. The slides were covered, sealed with Vectashield (Vector, Burlingame, CA, USA), and observed under a confocal laser scanning microscope (FV1000; Olympus, Tokyo, Japan). The digital images were captured and processed using FV10-ASW 1.6 software (Olympus). The images obtained were used to document and analyze the ratio difference of double- or triple-labeled neuron numbers in the UAC and control groups (*n* = 6). The total number of cell bodies counted in each rat was obtained from 10 sections.

### RNA Extraction and Real-Time PCR Assay

The brainstem was removed after perfusion with 200 ml of 5 mM PBS (pH 7.3). Cryosections of 100 μm thickness were made at the level of the Vme. Then, Vme tissue was collected. RNA was isolated using Trizol and purified with the RNeasy Mini Kit. Gene expression was analyzed using the Applied Biosystems 7500 Real-time PCR machine. The primers for VGLUT1 gene are GAGTCACCTGCACTACACCC (forward), TGAGGAACACGTACTGCCAC (reverse; GenBank accession no.: NM_053859.2). Target mRNA levels were normalized and are displayed as fold changes compared to those of the control group.

### Western Blot

Brain tissue from UAC and control mice was placed in cold PBS, and dissection was performed, with the aid of prominent landmarks, to obtain DMV nuclei. The pancreas was also isolated from the same mouse. The brain and pancreas samples were homogenized in extraction buffer, 20 mM Tris-HCl (pH 7.4), 5 mM ethylenediaminetetraacetic acid (EDTA), 140 mM sodium chloride (NaCl), 1% Triton X-100, 1 mM sodium orthovanadate (Na_3_VO_4_), 1 mM phenylmethanesulfonyl fluoride (PMSF), 10 mM sodium fluoride (NaF), and 1 mg/ml aprotinin at 4°C. The extracts were centrifuged at 12,000 × g for 20 min at 4°C. The concentration of protein in the supernatant of each sample was measured using the Bio-Rad Protein Assay kit (Bio-Rad, Hercules, CA, USA); thereafter, protein was denatured by incubation at 95°C for 5 min in SDS-loading buffer. Proteins were fractionated by SDS-PAGE and transferred onto an Immobilon-P membrane (Millipore). The membranes were blocked with 5% non-fat milk for 2 h and incubated overnight at 4°C with primary antibodies. The protein from the nuclei was incubated with mouse anti-β-actin (1:2,000; Santa Cruz Biotechnology, Santa Cruz, CA, USA) and mouse anti-VGLUT1 (1:500; Millipore). The protein from the pancrea was incubated with rabbit anti-actin (1:2,000; SantaCruz Biotechnology) and mouse anti-AChE (1:500; Abcam, Cambridge, MA, USA). The blots were developed using ahorseradish peroxidase-conjugated secondary antibody and enhanced chemiluminescence detection. The immunolabeled band was detected by the enhanced chemiluminescence detection method (Amersham Pharmacia Biotech, Piscataway, NJ, USA) and exposure to film. The scanned images were quantified and analyzed with Image J software. Target protein levels were normalized against β-actin levels and expressed as fold changes relative to those of the control group at 3 weeks.

### Statistical Analysis

Data acquisition and analysis were performed by a researcher blinded to the study group assignment using SPSS 16.0 (SPSS Inc., IL, USA). Data are expressed as the mean ± standard deviation (SD). Statistical significance in the UAC group and control group at the same time point, evaluated by unpaired *t*-test, was used to make comparisons. For multiple comparisons between the age-matched three groups at different time points, respectively, one-way analysis of variance was applied, and Tukey's multiple comparisons test was used to compare between two groups at the same time point. The threshold for statistical significance was *P* < 0.05, *P* < 0.01, or *P* < 0.001, as specified.

## Results

### Vme Branched to Periodontal Tissues Peripherally and to DMV Centrally

**Figure 2:**
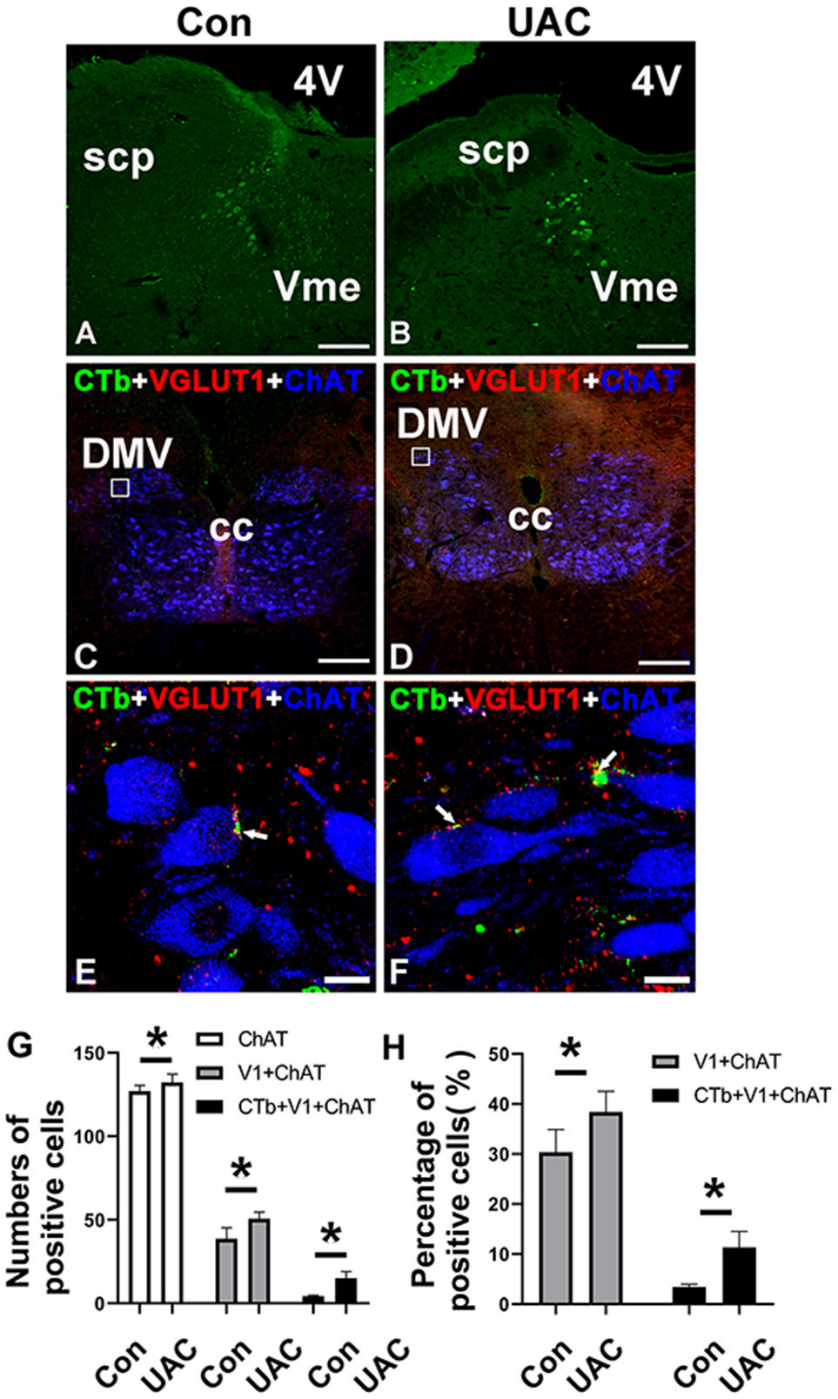
The expression of Cholera toxin B subunit (CTb) in the trigeminal mesencephalic nucleus (Vme) after being injected into the inferior alveolar nerve in the control (Con) groups **(A)** and unilateral anterior crossbite (UAC) **(B)**. Triple-immunofluorescence histochemistry for CTb (green), vesicular glutamate transporter 1 (VGLUT1) (red), and choline acetyltransferase (ChAT) (blue) immunoreactivities in a transverse section through the dorsal motor nucleus of vagus nerve (DMV) **(C–F)** from CTb-injected rats. In UAC group, the rats were exposed to UAC for 8 weeks, which was started from 6-weeks old. The framed areas in **(C)** and **(D)** are magnified in **(E)** and **(F)**. White arrows indicate axonal buttons dually labeled with VGLUT1 (red) and CTb (green) that are merged (yellow), and are in close apposition to ChAT-immunopositive DMV soma (blue). 4V, fourth ventricle; cc, corpus callosum; scp, superior cerebellar peduncle. Scale bar = 200 μm in **(A–D)**; 10 μm in **(E,F)**. The average number **(G)** and the percentage **(H)** of ChAT/CTb/VGLUT1 triple-labeled neurons in DMV were significantly different between aforementioned two groups (**G,H**; *P* < 0.05). The average number **(G)** and the percentage **(H)** of ChAT/VGLUT1 double-labeled neurons in DMV were also significantly different between aforementioned two groups (**G,H**; *P < 0.05).

To identify the presence of Vme–DMV circuit, CTb was injected into the inferior alveolar nerve of rats as we previously reported (Liu et al., 2017, 2018). Many cell bodies were found to be labeled retrogradely with CTb in the Vme of both UAC and Con group (Figures 2A,B). Immunofluorescence staining indicated that in DMV of both UAC and Con groups, there were quite a couple of the CTb-labeled axonal profiles (Figures 2C,D). When observed under higher magnification, CTb and VGLUT1 double-labeled axonal profiles were in close apposition to DMV neuronal profiles labeled by ChAT (Figures 2E,F).

In the UAC group, the average number of ChAT/CTb/VGLUT1 triple labeled neurons in DMV was 15.00 ± 4.00, accounting for 11.35 ± 3.16% of the number of ChAT positive neurons; while in the control group, they were 4.33 ± 0.57 and 3.42 ± 0.56%, respectively. The differences between the two groups were significant (Figures 2G,H; *P* < 0.05, all). In the UAC group, the average number of ChAT/VGLUT1 double-labeled neurons in DMV was 50.67 ± 4.04, accounting for 38.38 ± 4.14% of the number of ChAT positive neurons; while in the control group, they were 38.67 ± 6.66 and 30.37 ± 4.52%, respectively. The differences between the two groups were significant (Figures 2G,H; *P* < 0.05, all).

### UAC Stimulated the mRNA Expression of VGLUT1 in Vme and Protein Expression of VGLUT1 and ChAT in DMV

**Figure 3:**
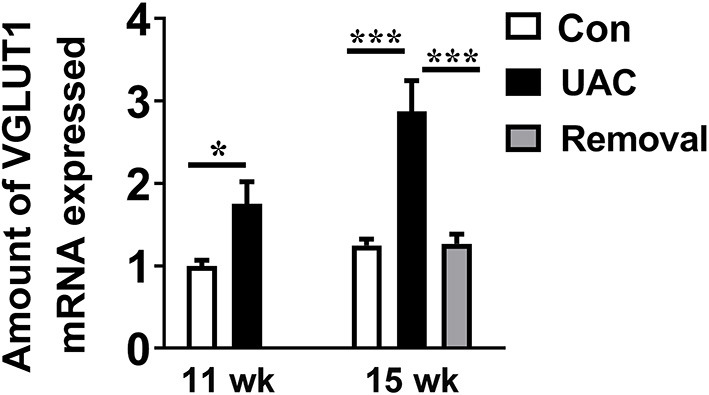
Comparisons of the levels of vesicular glutamate transporter 1 (VGLUT1) mRNA expressed in neurons in the trigeminal mesencephalic nucleus (Vme) of mice, detected by real-time polymerase chain reaction (PCR), between the unilateral anterior crossbite (UAC) and the sham-operation control (Con) groups at 11 weeks, and between UAC, control and removal groups at 15-weeks. VGLUT1 mRNA expression in Vme neurons showed that VGLUT1 mRNA levels, normalized to the GAPDH expression, were significantly changed as times of fold compared to those mRNA of the control group at 11 weeks (*P* < 0.05 in 11 weeks, *P* < 0.001 in 15 weeks). VGLUT1mRNA expression levels in the Removed rats were significantly lower than that in the UAC cases at time points of 15 weeks (*P* < 0.001). *n* = 6. Data are presented as mean ± standard deviation. *P < 0.05, ***P < 0.001.

**Figure 4:**
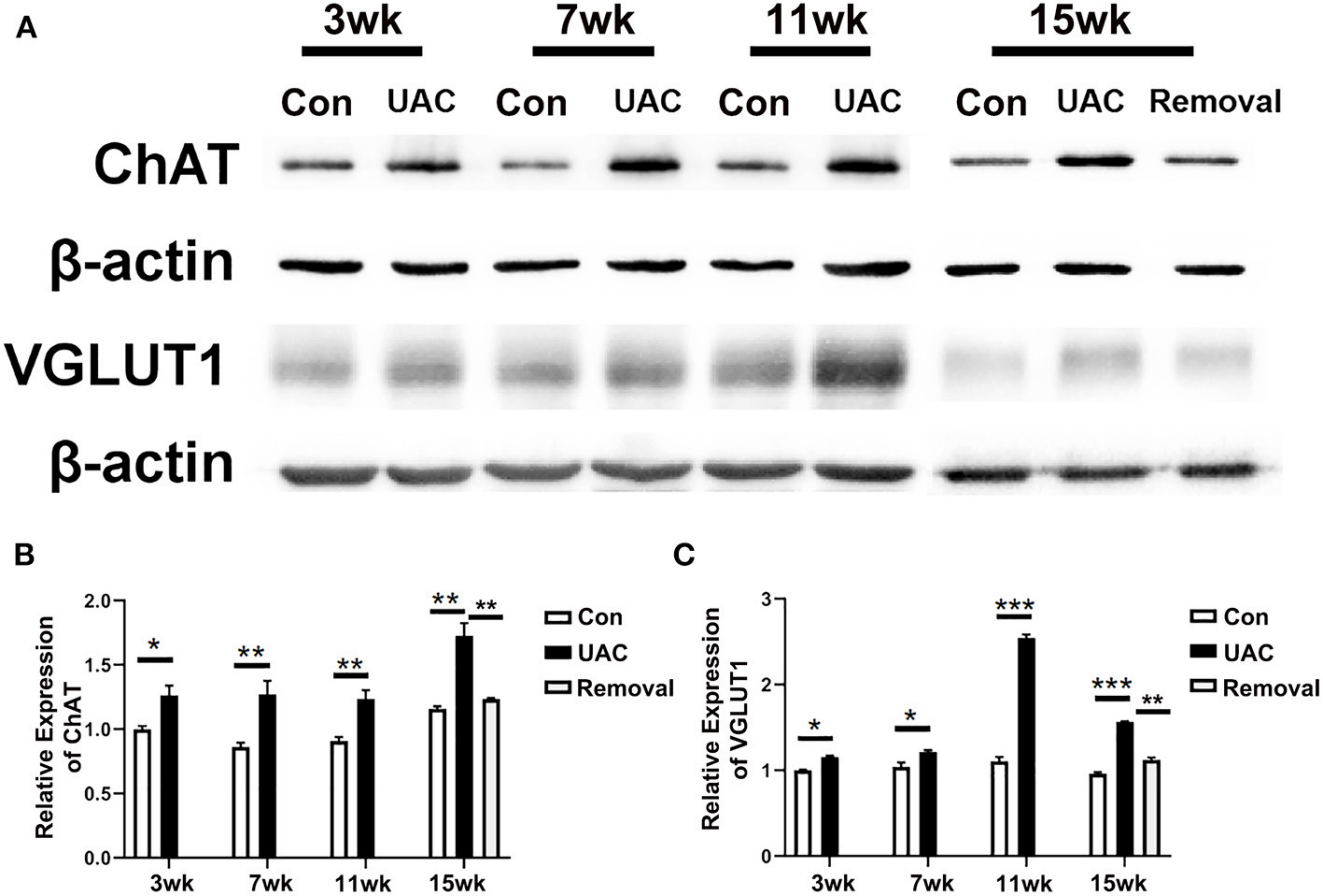
Comparison of the protein expression of vesicular glutamate transporter 1 (VGLUT1) and choline acetyltransferase (ChAT) in the dorsal motor nucleus of vagus nerve (DMV) of mice as revealed by Western blot assay **(A)** at 3-, 7-,11-, 15-weeks between Con, UAC, and Removal groups. Western blotting analysis displayed that protein levels of VGLUT1 and ChAT in the DMV of UAC cases were obviously higher than that in the control rats at different time points **(B,C)**. Statistical comparison of the blotting data unveiled that VGLUT1 and ChAT protein levels in the UAC rats were significantly higher than that in the control cases at 3-, 7-, 11-, 15-weeks (*P* < 0.05 at 3 weeks, *P* < 0.01 at 7-, 11-, 15-weeks in VGLUT1 expression; *P* < 0.05 at 3, 7 weeks, *P* < 0.001 at 11, 15 weeks in ChAT expression). VGLUT1 and ChAT protein levels in the Removed rats were significantly lower than that in the UAC cases at time points of 15 weeks (*P* < 0.01). Protein level change is exhibited as times of fold against the control group at 3 weeks. Con, sham-operation control group; UAC, the group in which the mice were exposed to unilateral anterior crossbite; and Removal, the group in which UAC was installed to the mice for 11 weeks from 6-weeks old and then was withdrawn until being sampled at 15-weeks. *n* = 6. Data are presented as mean ± standard deviation. *P < 0.05, **P < 0.01, ***P < 0.001.

To detect whether the Vme–DMV circuit was activated by UAC, the mRNA expression of VGLUT1 in Vme, the protein expression of VGLUT1 and ChAT in DMV were tested. Quantitative real-time PCR data indicated that the levels of VGLUT1 mRNA in Vme of UAC subgroups were markedly increased compared with the levels in Con groups. Removal of UAC at 15 weeks reversed the increase of VGLUT1 mRNA in Vme (Figure 3, *P* < 0.05 at 11 weeks, *P* < 0.001 at 15 weeks**)**. Data from Western blot of DMV at 3-, 7-, 11-, and 15-weeks (Figure 4A) confirmed the upregulated expression of VGLUT1 and ChAT proteins in the UAC group compared with those in the Con group (Figures 4B,C; *P* < 0.05 at 3 weeks, *P* < 0.01 at 7, 11, 15 weeks in VGLUT1 expression; *P* < 0.05 at 3, 7 weeks, *P* < 0.001 at 11, 15 weeks in ChAT expression). Removal of UAC at 15-weeks reversed the UAC stimulated increase of VGLUT1 and ChAT proteins in DMV (Figures 4B,C; *P* < 0.01).

### UAC Up-Regulated AChE Expression in Pancreas

**Figure 5:**
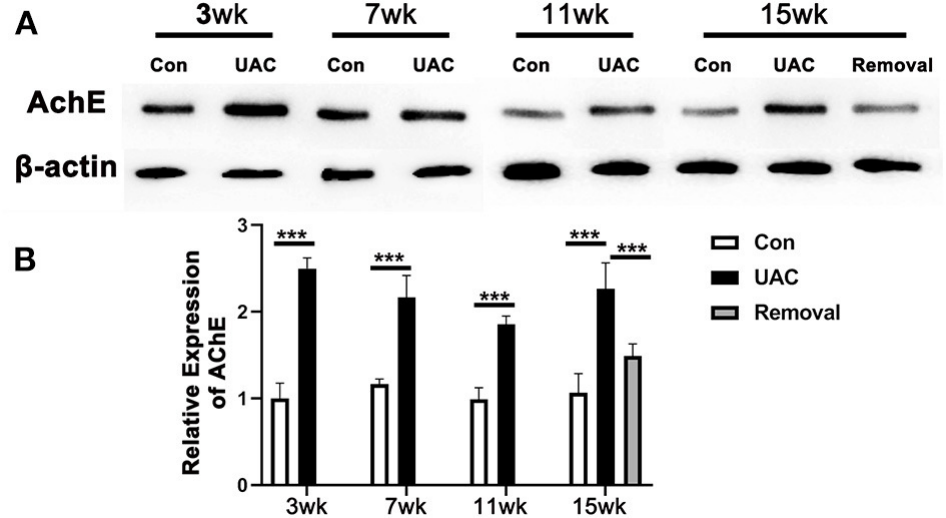
Comparison of the acetylcholinesterase (AChE) protein expression level in pancreas as revealed by Western blotting assay **(A)** at 3-,7-,11-, and 15-weeks between Con, UAC, and Removal groups of mice. Changes exhibited as times of fold against the control group **(B)**. Statistical comparison of the blotting data unveiled that AChE protein levels in the UAC rats are significantly higher than that in the control cases at time points of 3, 7, 11, 15 weeks (*P* < 0.001, all). AChE protein levels in the Removed rats were significantly lower than that in the UAC cases at time points of 15 weeks (*P* < 0.001). Con, sham-operation control group; UAC, the group in which the mice were exposed to unilateral anterior crossbite; and Removal, the group in which UAC was installed to the mice for 11 weeks from 6 weeks old and then was withdrawn until being sampled at 15-weeks. *n* = 6. Data are presented as mean ± standard deviation. ***P < 0.001.

To detect the impact of the activated DMV on pancreas, the protein expression of AChE in pancreas tissues was tested. Western blot data showed that the protein expression level of AChE in pancreas was increased in UAC groups compared with the Con group and the increased expression was partially attenuated in the 15-weeks removal group (Figures 5A,B; *P* < 0.001).

### UAC Up-Regulated Glucagon and Insulin Expression in Islet and Serum, and Increased the Postprandial Blood Glucose Level

**Figure 6:**
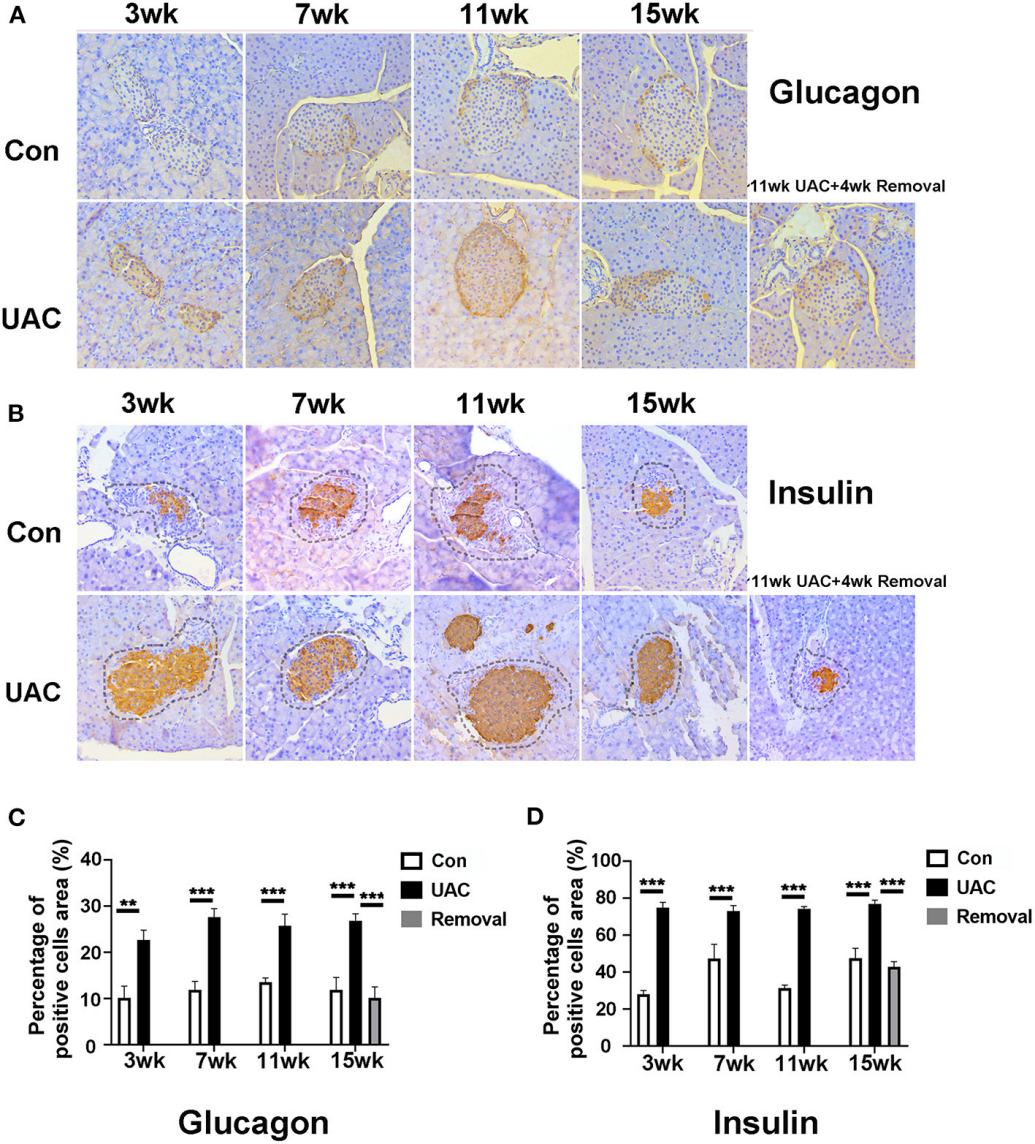
Comparison of the glucagon **(A)** and insulin **(B)** expression in islets at 3-,7-,11-, and 15-weeks between Con, UAC, and Removal groups. Changes exhibited as times of fold against the control group at 3 weeks **(C,D)**. The insulin and glucagon-positive cell area was stained orange and other negative cell area of islet was stained pale. The dashed lines in Figure 5B represented the approximate outline of the islets. Statistical comparison of the insulin and glucagon-positive cell area unveiled that insulin and glucagon level in the UAC rats were significantly higher than that in the control cases at time points of 3, 7, 11, 15 weeks (*P* < 0.01 at 3 weeks, *P* < 0.001 at 7, 11, 15 weeks in glucagon expression; *P* < 0.001 in insulin expression). Insulin and glucagon levels in the Removed rats were significantly lower than that in the UAC cases at time points of 15 weeks (*P* < 0.001). Con, sham-operation control group; UAC, the group in which the mice were exposed to unilateral anterior crossbite; and Removal, the group in which UAC was installed to the mice for 11 weeks and then was withdrawn until being sampled at 15-weeks. *n* = 6. Data are presented as mean ± standard deviation. **P < 0.01, ***P < 0.001.

To demonstrate the effect of DMV on pancreas function, immunohistochemistry detection for pancreas was performed. Upregulation of glucagon (Figure 6A) and insulin (Figure 6B) expression level was obvious in islet of UAC group compared with the age matched Con group. The percentages of glucagon and insulin positive cells areas were higher in UAC group than Con group (Figures 6C,D; *P* < 0.01 at 3 weeks, *P* < 0.001 at 7, 11, 15 weeks in glucagon expression; *P* < 0.001 in insulin expression). The increased expression was partially attenuated in the 15-weeks Removal group (Figures 6C,D; *P* < 0.001).

**Figure 7:**
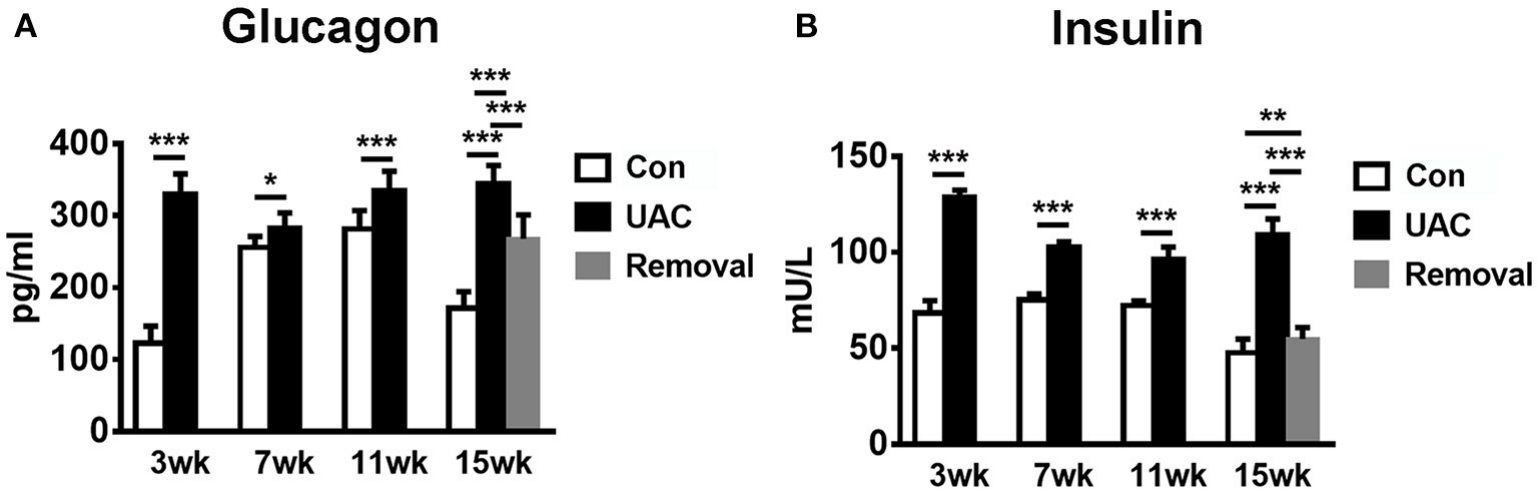
Comparison of the glucagon and insulin level in serum **(A,B)** as revealed by the ELISA assay at 3-, 7-, 11-, and 15-weeks between Con, UAC, and Removal groups. Statistical comparison unveiled that glucagon and insulin level in the UAC rats were significantly higher than that in the control cases at time points of 3, 7, 11, 15 weeks (*P* < 0.05 at 7 weeks, *P* < 0.001 at 3, 11, 15 weeks in glucagon expression; *P* < 0.001 in insulin expression). Glucagon and insulin levels in the Removed rats were significantly lower than that in the UAC cases at time points of 15 weeks (*P* < 0.001 in glucagon expression; *P* < 0.01 in insulin expression). Con, sham-operation control group; UAC, the group in which the mice were exposed to unilateral anterior crossbite; and Removal, the group in which UAC was installed to the mice for 11 weeks and then was withdrawn until being sampled at 15-weeks. *n* = 6. *P < 0.05, **P < 0.01, ***P < 0.001.

**Figure 8:**
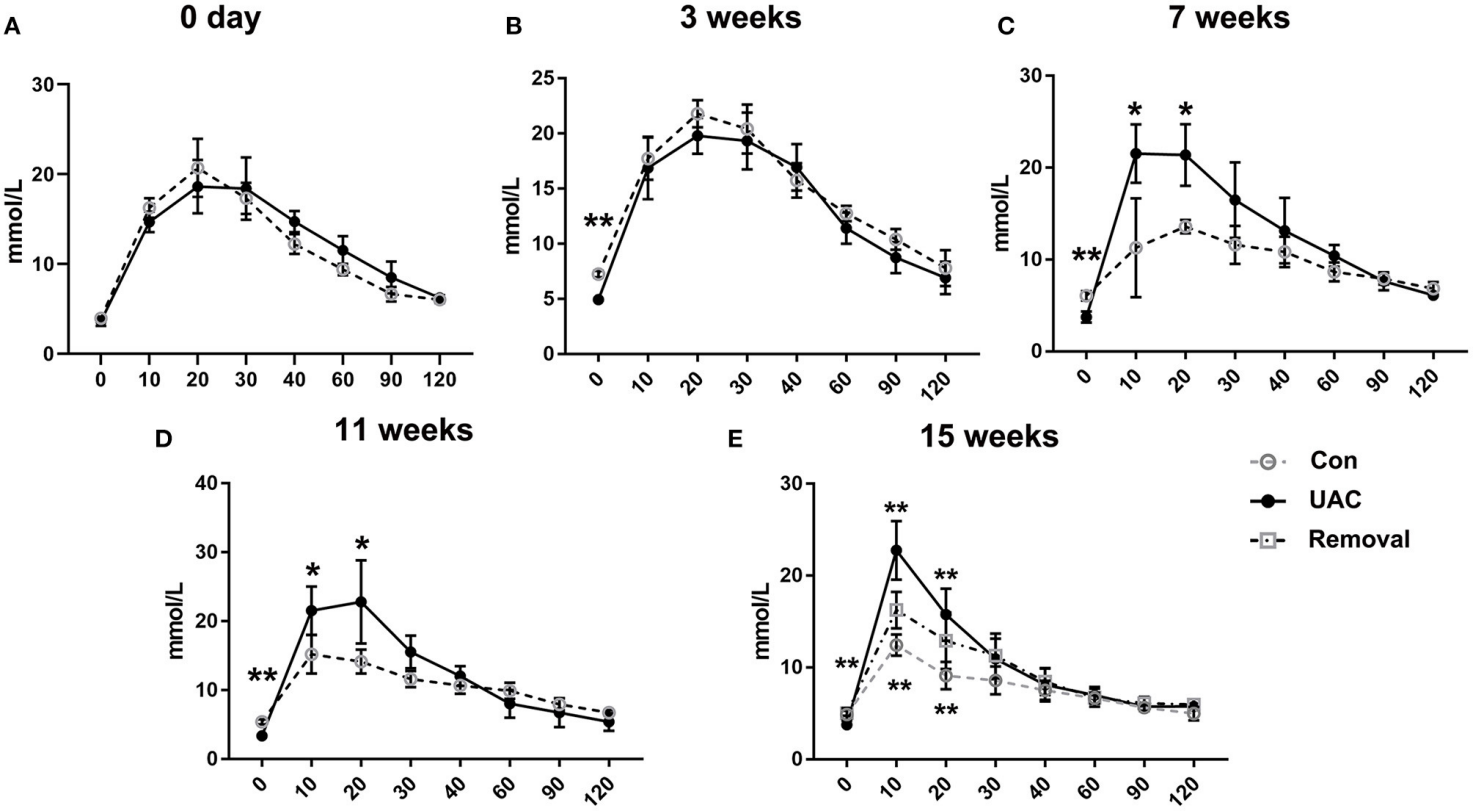
Comparison of the fasting blood glucose level and the postprandial blood glucose level detected by the oral glucose tolerance test (OGTT) assay at 3-, 7-, 11-, and 15-weeks between Con, UAC, and Removal groups. Statistical comparison of the OGTT results **(A–E)** showed that the fasting blood glucose of UAC group was lower in 3-, 7-, 11-, and 15-weeks than that in the age matched Con group **(B–E**, *P* < 0.01). The postprandial blood glucose was increased at 10 and 20 min after administration of the glucose solution in 7-, 11-, and 15-weeks **(C–E**, *P* < 0.05), although that in 0 day and 3-weeks was identical to that of Con subgroups **(A,B)**. Postprandial blood glucose level in the Removed rats were significantly lower than that in the UAC cases at time points of 15 weeks (**E**, *P* < 0.01). Con, sham-operation control group; UAC, the group in which the mice were exposed to unilateral anterior crossbite; and Removal, the group in which UAC was installed to the mice for 11 weeks from 6-weeks old and then was withdrawn until being sampled at 15-weeks. *n* = 6. *P < 0.05, **P < 0.01.

To confirm the promoted pancreas function by UAC, serum detection was conducted. ELISA data demonstrated that the glucagon and insulin levels in serum were upregulated in the UAC rats compared with the control cases at time points of 3, 7, 11, 15 weeks (*P* < 0.05 at 7 weeks, *P* < 0.001 at 3, 11, 15 weeks in glucagon expression; *P* < 0.001 in insulin expression). The increased expression was partially attenuated in the 15-weeks Removal group (*P* < 0.001 in glucagon expression; *P* < 0.01 in insulin expression; Figures 7A,B). The OGTT results (Figures 8A–E) showed that the fasting blood glucose of UAC group was lower in 3-, 7-, 11-, and 15-weeks than that in the age matched Con group (Figures 8B–E; *P* < 0.01). The postprandial blood glucose was increased at 10 and 20 min after administration of the glucose solution in 7-, 11-, and 15-weeks (Figures 8C–E; *P* < 0.05), although that in 0 day and 3-weeks was identical to that of Con subgroups (Figures 8A,B). All the changes were significantly attenuated in the removal group (Figure 8E, *P* < 0.01).

## Discussion

**Figure 9:**
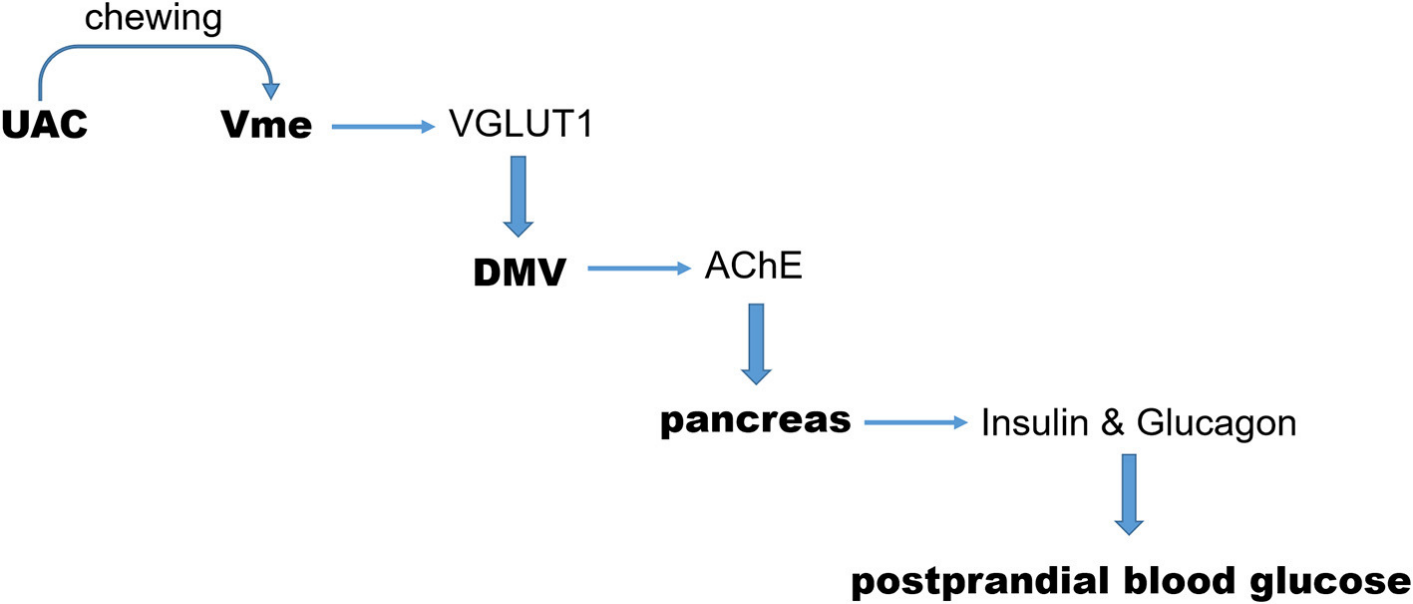
Sketch of the present story. During chewing function, the excitatory message of UAC on Vme and then on DMV, mediated by VGLUT1, was delivered to pancreas *via* AChE. In this way, UAC stimulated the secretion of insulin and glucagon which send an impact on the postprandial blood glucose level. UAC, unilateral anterior crossbite; Vme, the trigeminal mesencephalic nucleus; DMV, The dorsal motor nucleus of vagus nerve; VGLUT1, vesicular glutamate transporter 1.

As we indicated in the introduction section, the sign of CTb positivity in neurons of the Vme in animals accepted CTb injection into the alveolar nerve was taken as an indication of the afferent message from the mandibular periodontal region where the proprioceptive receptors are located at (Liu et al., 2017). And the increase of VGLUT1 protein in the Vme-targeted neurons was taken as a sign of excitatory impact of Vme on that nucleus (Pang et al., 2006). By using these reported methods, presently, we demonstrated that the CTb labeled VGLUT1 positive axon terminals of Vme were distributed surrounding neurons in DMV. The quantitative results of ChAT/CTb/VGLUT1 triple-labeled neurons suggest that DMV in UAC group received more excitatory message from Vme. Such an effect was supported by the increased VGLUT1 protein levels in DMV with the method of Western blot assay. Further, the AChE expression in pancreas was stimulated, the levels of insulin and glucagon in both pancreas and serum were upregulated, and the postprandial blood glucose was increased in UAC mice. Removal of UAC reversed not only the VGLUT1 mRNA expression in Vme, but also the VGLUT1 protein expression in DMV, AChE protein expression in pancreas, insulin and glucagon expression in pancreas and serum, and postprandial blood glucose level. In other word, as we described in Figure 9, UAC activated Vme–DMV circuit and send impact on pancreas function. When the UAC stimulated excitatory activity in Vme was down-regulated by removal of UAC, the excitatory impact of Vme on DMV and pancreatic secretory function was attenuated.

Pancreatic hormones, typically insulin and glucagon, are discussed widely in control of blood glucose level. When insulin is deficient, the amount of glucose entering the tissue cells is reduced, which causes the liver to release glucose into the blood to increase the blood glucose level. That is the mechanism of the increase of renal glucose level in diabetes patients. Impaired glucagon secretion predisposes some patients with type 1 diabetes mellitus to hypoglycemia; whereas hyperglycemia in patients with type 1 diabetes mellitus or type 2 diabetes mellitus is often associated with hyperglucagonemia (Campbell and Drucker, 2015). Dorsal motor nucleus of vagus nerve, the important visceral motor nucleus, takes a primary role in the control of pancreatic exocrine secretion (Li et al., 2006). Our present data demonstrated the dental impact on the DMV modulated pancreatic exocrine secretion because both insulin and glucagon levels were increased in UAC model and were reversed in UAC Removal group.

Even though the present fasting blood glucose level was down-regulated in UAC group which agreed well with the higher level of insulin, and the postprandial blood glucose level was up-regulated in UAC group which in accordance with the higher level of glucagon, the timeline contradict causal correlation is obvious. Choline acetyltransferase expression shows a constant upregulation, VGLUT1 expression peaks at 11 weeks in the DMV, while the biggest changes in pancreatic AChE, and insulin and glucagon in islets seem to occur at the 3-weeks time point. The increased average number of ChAT/VGLUT1 double-labeled neurons, especially in CTb-negative areas, indicated that DMV also received VGLUT1 positive innervation from other nuclei than the Vme. Clearly, the modulation of other factors on DMV in UAC treated animals should not be neglected.

It has been indicated that the DMV receives myelinated and unmyelinated vagal afferent input from the periphery, and also afferent input from central nervous system structures, including the insular cortex, the central nucleus of the amygdala, the paraventricular hypothalamic nucleus, the lateral hypothalamic area, the dorsomedial hypothalamic nucleus, the posterior hypothalamus, the mesencephalic central gray matter, the parabrachial nucleus, the medullary reticular formation, and the raphe obscurus nucleus (ter Horst et al., 1984). Endogenously occurring excitatory (glutamate) and inhibitory amino acids (GABA) could have a marked influence on DMV vagal output to the pancreas, respectively. The pancreatic secretion can be mediated by gastrointestinal hormones and vagovagal reflex pathways synergistically (Mussa and Verberne, 2013). Ghrelin, a preventer of life-threatening falls in blood glucose, has also been reported to decrease glucose-induced insulin release and increase glucose level (Alamri et al., 2016). Leptin deficiency is associated with insulin resistance and impaired glucose metabolism (do Carmo et al., 2019). Leptin administration plays powerful anti-diabetic actions that improve tissue glucose uptake/oxidation and reduce hepatic glucose output (da Silva et al., 2006, 2020). The impact of cortisol on serum insulin levels has been widely discussed (Stolk et al., 1996). The lateral habenula, which is involved in a set of depressive symptoms, has been well-recognized as a negative regulator of the mono-aminergic systems in the central nervous system. In our recent report, we indicated that UAC increased neuronal activity in the LHb and produced anxiety in UAC rats, implying an induced depression (Liu et al., 2019). Depression is associated with cross-sectional and longitudinal alterations in the diurnal cortisol curve, including a blunted cortisol awakening response and flattening of the diurnal cortisol curve, the latter of which is associated with insulin resistance and type 2 diabetes mellitus (Lam et al., 2009; Joseph and Golden, 2017).

Recent scientific publications support the role of postprandial glucose as a key contributor to overall glucose control and a predictor of microvascular and macro-vascular events (Madsbad, 2016). Evidence supports postprandial glucose control as an important strategy in the comprehensive management plan of individuals with diabetes (Madsbad, 2016). The complexity of postprandial glucose patterns and the effects of dietary fat, protein, and glycemic index on acute postprandial glucose control in type 1 diabetes has been deeply discussed (Bell et al., 2015). Other dietary factors, such as vinegar, have been also reported to impact postprandial glucose and insulin levels (Shishehbor et al., 2017). The Roux-en-Y gastric bypass surgery which is usually take as a modality to improve glucose control in patients with type 2 diabetes had been reported to develop a life-threatening complication of hyperinsulinemic hypoglycemia (Honka and Salehi, 2019). Our present data, as far as our extension, for the first time, proposed the dental occlusion as a biomechanical factor in upregulation of the postprandial blood glucose level.

In summary, our present data propose Vme–DMV circuit through the activation of which the aberrant occlusion elicits an increased postprandial glucose level as a trigeminal neuroendocrine response. If the role of the neuronal circuit between Vme and DMV in the effect of insulin, glucagon and blood glucose are confirmed in clinic, new approaches to occlusion diagnosis and therapeutic intervention are expected to be developed to prevent harmful endocrine response in patients with dental problem.

## Data Availability Statement

The datasets presented in this study can be found in online repositories. The names of the repository/repositories and accession number(s) can be found in the article/supplementary material.

## Ethics Statement

The animal study was reviewed and approved by The Animal Care and Use Committees of the Fourth Military Medical University.

## Author Contributions

XinL, MS, JL, and MW conceived and designed the experiments. XinL, MS, HR, and MX performed the experiments and acquired and analyzed the data. CZ, DW, and XiaL helped in analyzing data. XinL, MS, HR, JL, and MW wrote the article. All authors discussed, read, contributed to the article, and approved the submitted version.

## Conflict of Interest

The authors declare that the research was conducted in the absence of any commercial or financial relationships that could be construed as a potential conflict of interest.
